# Gender differences in segmental foot motions during gait using 3D multi-segment foot model

**DOI:** 10.1186/1757-1146-7-S1-A75

**Published:** 2014-04-08

**Authors:** Sang Gyo Seo, Dong Yeon Lee, Ji-Beom Kim, Seong Hyun Kim, Hye Sun Park, Hyo Jeong Yoo, Sung Ju Kim, Jihyeung Kim, Kyoung Min Lee, Chin Youb Chung, In Ho Choi

**Affiliations:** 1 Department of Orthopedic Surgery, Seoul National University Hospital, Seoul, Korea; 2 Department of statistics, Korea University, Seoul, Korea; 3 Department of Orthopedic Surgery, Seoul National University Boramae Medical Center, Seoul, Korea; 4 Department of Orthopedic Surgery, Seoul National University Bundang Hospital, Seongnam, Korea

## 

**Figure 1:**
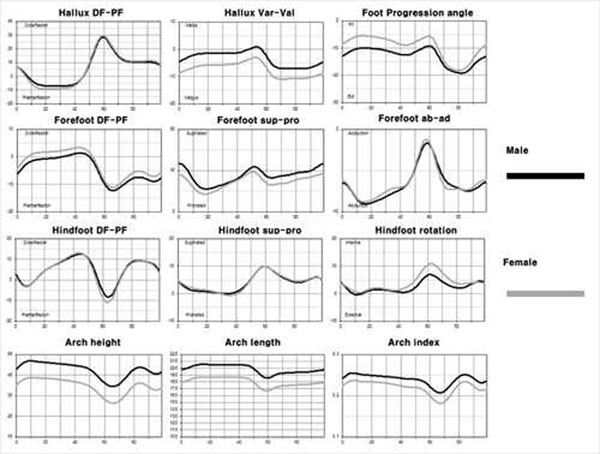
Comparison of the mean foot segmental motion between two genders. The both gender had similar patterns of segmental foot motions in spite of the gender differences in the specific motion of some segments.

There might be gender differences in segmental foot motion considering the gender differences in the foot shape and the prevalence of pathologies [1,2]. The objectives of this study were 1) to obtain reference data of segmental motion of the foot using a multi-segment foot model (MFM) with 15-marker set from healthy adults; 2) to find gender differences in segmental foot motion during gait. One hundred feet of 100 healthy adults (50 males, 50 females) with 20-35 years old were tested by Cleveland Clinic marker set and six additional foot markers. We presented demographic data of participating subjects. Females were shorter, both in height and length. Hallux valgus angle on static status was significantly higher in female. Talo-1^st^ metatarsal angle was not significantly different. The cadence (steps/min) was significantly more frequent in female than in male. The stride length, the step width, and the step time were significantly longer in male. The speed and the proportion of stance phase were not significantly different (Table 1). The range of segmental motion (hallux, forefoot, hindfoot) and arch data were recorded during the gait and compared between male and female. The both genders had similar patterns of segmental foot motions. The range of sagittal motion and coronal angulation of the hallux was greater during gait in females. The range of motion on the hindfoot was also greater in females. The male had higher adjusted arch height and arch index. However, the range of adjusted arch height was larger in females (Figure 1). We demonstrated that there was a substantial temporal pattern of the foot segmental motion in normal adults. We also presented that there was a significant gender difference the motion of specific foot segment. We believe that data from this study might be used as a reference data to evaluate the effect of certain condition on the segmental motion of the foot and to reveal the gender difference in prevalence and prognosis of foot and ankle pathologies.

**Table 1:** Basic gait parameters

	Male (mean ± SD)	Female (mean ± SD)	p- value
Cadence (cm)	110.3 ± 5.7	116.4 ± 6.5	< 0.001
Speed (cm/sec)	123.3± 8.9	124.9± 7.5	0.445
Stride length (cm)	133.9± 7.3	128.3± 7.1	< 0.001
Step width (cm)	66.9 ± 3.7	64.1 ± 3.6	< 0.001
Proportion of stance phase (%)	59.7 ± 1.2	59.3 ± 0.8	0.123
